# Characterization of the complete plastome of *Spiraea trilobata* (Rosaceae), a perennial shrub

**DOI:** 10.1080/23802359.2021.2018948

**Published:** 2022-01-24

**Authors:** Hao Qin, Xinxin Zhu, Xin Zhang, Xuejie Zhang, Luoyan Zhang

**Affiliations:** Shandong Provincial Key Laboratory of Plant Stress Research, College of Life Sciences, Shandong Normal University, Ji’nan, Shandong, China

**Keywords:** Spiraea trilobata, complete plastome, phylogeny

## Abstract

The complete plastome of *Spiraea trilobata*, a shrub, is determined. The plastome is 155,981 bp in length and comprises a large single-copy region (84,417 bp), a small single-copy region (18,878 bp), and a pair of inverted repeats regions (26,343 bp). A total of 113 unique genes are annotated for the plastome of *S. trilobata*, containing 79 protein coding genes (PCGs), 30 tRNAs, and four rRNAs. The GC content of this plastome is 36.8%. Phylogenomic analysis based on 17 plastomes reveals that *S. trilobata* is sister to *Spiraea blumei*. Compared with *S. blumei*, *S. trilobata* lacks both *trnM-CAU* and *ycf1*. In addition, the GC content of both species is the same.

*S. trilobata* belongs to *Spiraea* in Spiraeoideae of Rosaceae. It is mainly distributed in China, Russia and Turkey (Yu and Kuan 1963) and often grows in the shrubs on rocky slopes, forest margins, roadsides and ditches with an altitude of 450–2400 m (Huo et al. 2019). It has strong adaptability and is the dominant species in poor soil (Liu et al. 2017). It has beautiful flowers and delicate leaves, so it is a common ornamental shrub in garden and has important horticultural and economic value (Yu et al. 2018). The leaves and fruits of *S. trilobata* have the functions of promoting blood circulation, removing blood stasis, detumescence and relieving pain (Olennikov and Chirikova 2018). However, the complete plastome of *S. trilobata* has not been reported. In this study, we showed that the plastome of *S. trilobata*, which would be helpful for species identification and the phylogenetic analysis of the Rosaceae.

Silica-dried leaves of *S. trilobata* were collected from Lushan Forest Park (Shandong, China; 23°32′ N, 118°6′ E). The voucher specimen (XLC47) was deposited at College of Life Sciences, Shandong Normal University. Modified CTAB method was used for plant total DNA extraction (Doyle and Doyle 1987; Guo et al. 2020). The total genomic DNA was used for library preparation and paired-end (PE) sequencing by the Illumina MiSeq instrument at Novogene (Beijing, China). The plastome was assembled using getorganelle (Jin et al. 2020). Annotation was performed with PGA-Plastid Genome Annotator (Qu et al. 2019), coupled with manual correction using Geneious v8.0.2 (https://www.geneious.com). To determine the phylogenetic placement of *S. trilobata*, a maximum likelihood (ML) tree was reconstructed using RAxML v8.2.10 (Stamatakis 2014), including tree robustness assessment using 1000 rapid bootstrap replicates with the GTRGAMMA substitution model, based on the alignment of 79 shared PCGs using MAFFT v7.313 (Katoh and Standley 2013).

The complete plastome of *S. trilobata* (GenBank accession number: MW822176) is a circular molecular of 155,981 bp in length, consisting of a large single-copy region (84,417 bp), a small single-copy region (18,878 bp), and a pair of inverted repeats regions (26,343 bp). It encodes 113 unique genes, including 79 PCGs, 30 tRNAs, and four rRNAs. The GC content of this plastome is 36.8%. Phylogenomic analysis based on 17 plastomes reveals that *S. trilobata* is sister to *Spiraea blumei* (Figure 1). By comparing the plastomes of *S. trilobata* and *S. blumei*, we find that *S. trilobata* lacks two genes *trnM-CAU* and *ycf1*. The GC content of both species is the same.

**Figure 1.**
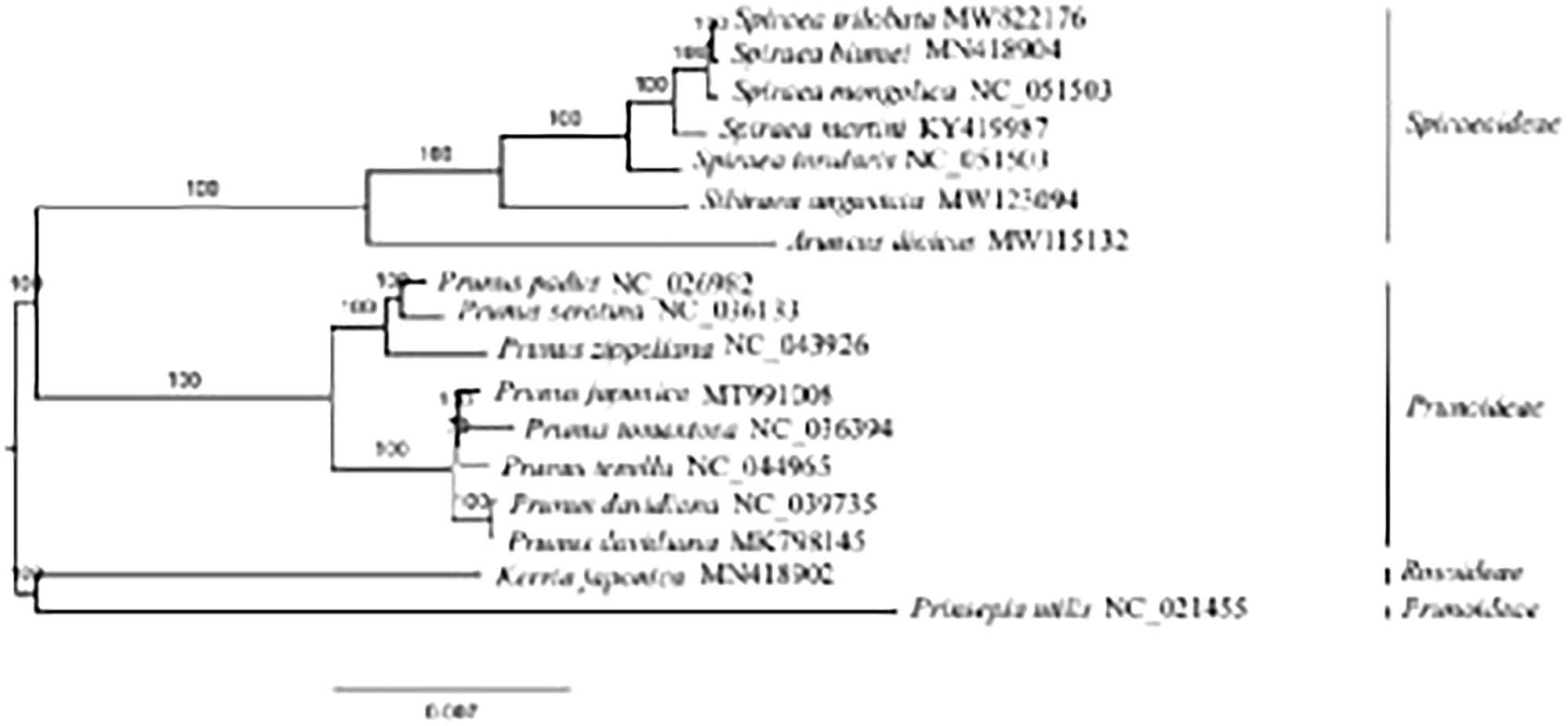
The maximum likelihood (ML) tree was reconstructed by 79 plastome genes. *Kerria japonica* and *Prinsepia utilis* were used as outgroups. The numbers on branches are bootstrap support values.

## Data Availability

The data that support the findings of this study are openly available in GenBank of NCBI at https://www.ncbi.nlm.nih.gov, reference number MW822176. The associated BioProject, SRA, and Bio-Sample numbers are PRJNA720486, SRR14180363, and SAMN18651951, respectively.
